# Langerhans cell histiocytosis of the hip in children

**DOI:** 10.11604/pamj.2019.33.230.17956

**Published:** 2019-07-18

**Authors:** Zied Jlalia, Dhia Kaffel

**Affiliations:** 1 Pediatric Orthopedics Department, Kassab Institute of Orthopedic Surgery, Ksar Said, Tunisia; 2 Rheumatology Department, Kassab Institute of Orthopedic Surgery, Ksar Said, Tunisia

**Keywords:** Langerhans cell histiocytosis, hip, children

## Image in medicine

Eight year old girl was referred to our consultation for a lameness with a flessum of the left hip with fever (38.5°C). Biology showed an inflammatory syndroma (CRP: 29 mg/l). Pelvic X-ray showed an osteolysis of the roof of the left acetabulum with regular contour (A). Magnetic resonance imaging (MRI) showed tissue thinning and damage to the roof of the left acetabulum (B). A scanno-guided biopsy was performed, anatomopathological study confirmed the diagnosis of Langerhans cell histiocytosis. A discharge, with traction was done. The child has improved and biology has normalized. The radiograph of the hip after 1 year showed a filling of the gap (C). Langerha's cell histiocytosis is a proliferation of mononuclear phagocytic cells. It is also called eosinophilic granuloma. It is often located at the skull and spine. The localization at the hip represents only 8% of the cases it is especially supra-acetabular its spontaneous evolution is favorable.

**Figure 1:**
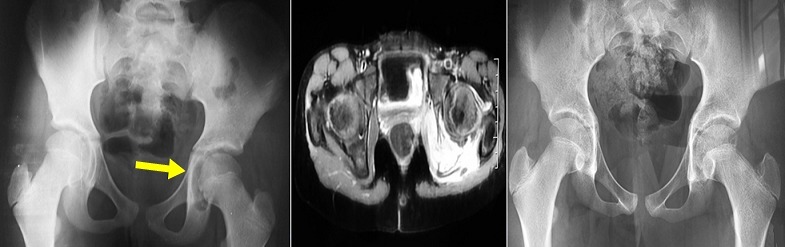
A) pelvic X-ray showed an osteolysis of the roof of the left acetabulum with regular contour; B) MRI of the hip joint showed tissue thinning and damage of the roof of the left acetabulum; C) pelvic X-ray after 1 year showed a filling of the gap

